# Prognostic value of diagnostic scales in community-acquired sepsis mortality at an emergency service. *Prognosis in community-adquired sepsis*

**DOI:** 10.1186/s12873-021-00532-1

**Published:** 2021-12-18

**Authors:** Jorge Clar, María Rosa Oltra, Raquel Benavent, Carolina Pinto, Adrian Ruiz, Maria Teresa Sanchez, Jose Noceda, Josep Redon, Maria Jose Forner

**Affiliations:** 1 Clinic Hospital. University of Valencia, 46010 València, Spain; 2 INCLIVA Research Institute, 46010 Valencia, Spain; 3 CIBERObn, Institute of Health Carlos III, Madrid, Spain

**Keywords:** Mortality, Prognosis, Sepsis, Community-acquired sepsis, Diagnostic scales

## Abstract

**Objectives:**

To asses the prognostic value of diagnostic scales in mortality of community-adquired sepsis and added value of additional parameters.

**Methods:**

Prospective observational study of patients with community-adquired sepsis in the Emergency Room of University Hospital. The study population were patients presented in the Emergency Room with confirmed infection and practicians sepsis diagnosis. Demographics, triage vital signs, inhaled oxygen fraction, inflammatory markers, biochemistry, all-cause mortality during hospitalization and three months after were recorded. Prognostic value of qSOFA, NEWS, SOFA, SIRS, and amplified scales were calculated by using logistic regression and ROC curves.

**Results:**

201 patients, 54% male, average age 77±11,2 years were included. Sixty-three (31.5%) died during hospitalization and 24 (12%) three months after discharge. At the time of admission vital signs related with in-hospital mortality were Glasgow Coma Scale <13, respiratory rate ≥22 bpm, temperature, oxygen desaturation, high flow oxygen therapy and heart rate. Patients dead in-hospital had lower PaCO2, higher lactate, glucose and creatinine. Greater predictive capacity of the scales, from higher to lower, was: qSOFA, NEWS2, SOFA and SIRS. Amplified scales with lactate >2mg/dl, glucose, blood level >190mg/dl and PaCO2 <35mmHg improved predictive value.

**Conclusion:**

Amplified-qSOFA and amplified-NEWS2 scales at Emergency Department may offer a better prognostic of septic patients mortality.

**Supplementary Information:**

The online version contains supplementary material available at 10.1186/s12873-021-00532-1.

## Highlights

- Sepsis is a high incidence disease with non-consensed scale for early diagnosis.

- Modification in commonly used scales can improvement their prognostic value.

- Amplified-qSOFA and amplified-NEWS2 may be useful tools for early sepsis diagnosis at Emergency Department.

## Introduction

Sepsis is a worldwide condition with high incidence and morbimortality. It is caused by a disregulated response of the organism to an infection and affects one million people every year [1]. According to the Third International Consensus Definition Task Force (Sepsis-3), sepsis is defined as a life-threatening organ dysfunction due to dysregulated host response to infection prefferibly suggested by a rapid increase of two points of the Sequential Organ Failure Assessment (SOFA) scale [2, 3]. Although actual sepsis incidence remains unknown, according to data published in Spain in 2014, three-hundred and thirty-three cases for every 100 000 habitants are estimated every year, some of them evolving to septic shock [4]. Among them, CAS is frequent being 10% of patients attending to Emergency Departments affected of infectious diseases. Moreover, 30-40% of septic patients in Intensive Care Units have the origen in CAS [4].

There are several quick diagnostic scales (qSOFA, SIRS, NEWS2) which were designed for early detection and management of septic patients, although there has been disagreement in the convenience of their use [5–15]. Likewise, contradictions about their prognostic value have been noticed, no only in community-acquired sepsis studies but also in studies based on specific groups of patients, critically ill [16], surgical [17], cirrhosis [18], oncologic [19]. Prognostic value of these scales is not consensual in community-adquired sepsis (CAS) [20, 21]. While some study reported that qSOFA had a prognostic accuracy for 28-day mortality comparable to SOFA and superior to SIRS, other studies did not identify risk scales as a prognostic marker of mortality. In a recent meta-analysis including 45 observational studies, qSOFA has been found to be a poorly sensitive predictive marker for in-hospital mortality [8]. The authors recommended develop another scoring system with higher value to identify high-risk of mortality patients with sepsis.

The aim of the present study is to assess the prognostic value of the sepsis diagnostic scales in CAS mortality, based on septic patients hospitalized in a University Hospital. We have assessed the predictive and prognostic value on mortality of each scale, both in a qualitative and quantitative approach. Furthermore, we have tested if modifying these scales, including new variables and cut-off points, can improve the prognostic value.

## Material and methods

**Fig. 1:**
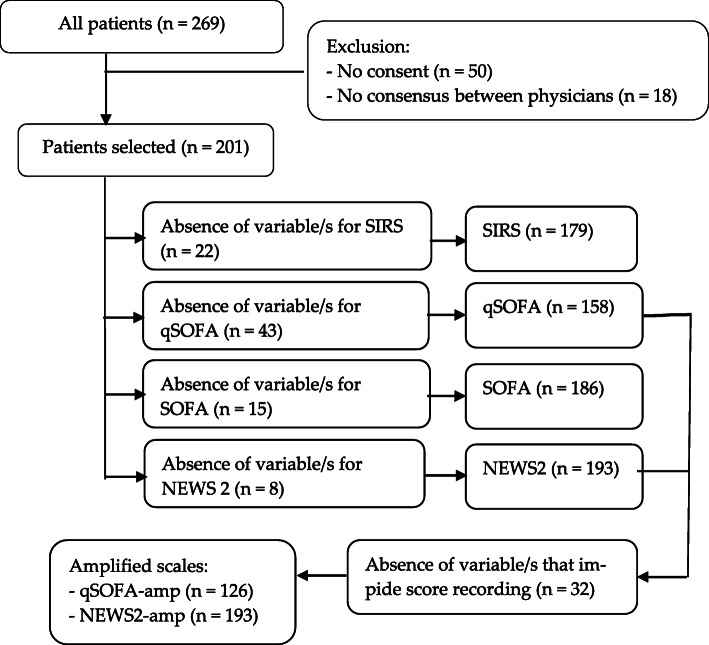
Flow chart

Observational prospective 8-month study performed from July 1st 2018 to February 28th 2019. Two hundred and one consecutive adult patients, from both genders and diagnosed of sepsis or septic shock, from the Emergency Room of the University Clinic Hospital of Valencia, Spain, were included in the study. The study population were patients presented in the Emergency Room with suspected or confirmed infection, excluding under 15-aged patients or nosocomial infections. Patients with suspected sepsis on values known at the time of ED encounter were included. Initially vital signs were recorded by ED nurses, and classify patients using the Manchester Triage System (MTS). Afterwards, subjects in which sepsis was confirmed by a physician based on symptoms and complementary tests (blood cultures, blood tests, image tests … ) during the hospitalization were included (Fig. 1 Flow chart). Data obtained during hospitalization and three months after hospital discharge were collected. This study was approved by Clinical University Hospital Ethical Committee and patients or a legal representative signed informed consent. Spanish Law 3/2018 of Data Protection and Guaranty of Digital Rights and corresponding European norms were followed.

### Recorded variables

From every patient the following data was obtained: a) age and gender; b) triage-recorded vital signs (respiratory rate, heart rate, blood pressure, Glasgow Scale, temperature (Celcius degrees tympanic measurement), oxygen saturation and inhaled oxygen fraction (FiO2). The first values, when the patient presents to the ED, blood count and serum biochemistry (glucose level, creatinine, urea and total bilirubin level), lactate and inflammatory response parameters RCP and procalcitonin. Mortality during hospitalization and until three months after hospital discharge was recorded.

### Risk scales

**Fig. 2:**
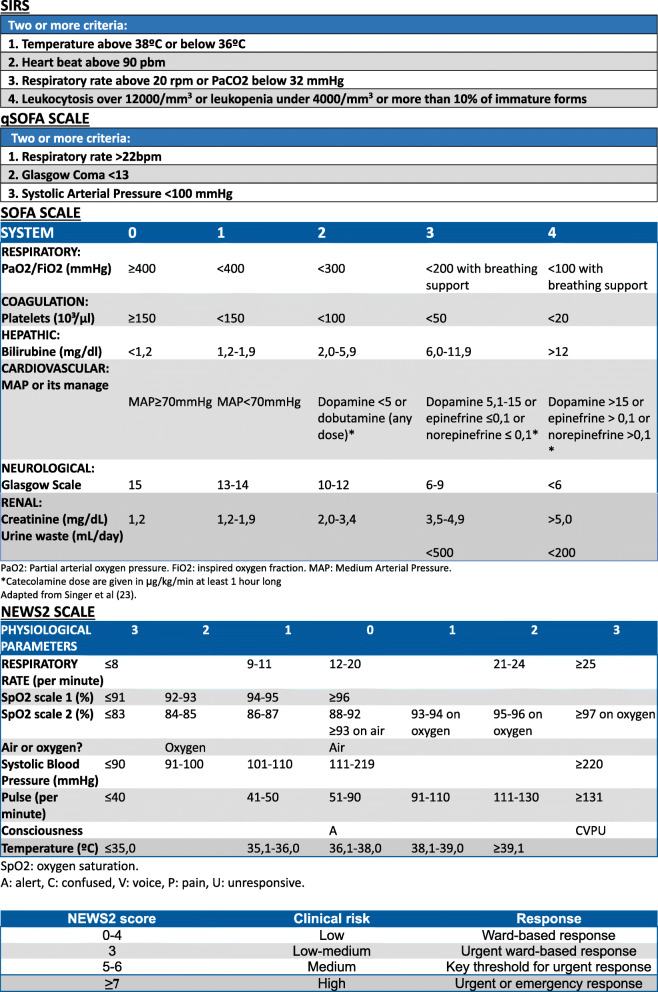
Sepsis scales

The scales whose prognostic value was calculated are shown in Fig. 2.

#### Systemic inflammatory response syndrome (SIRS)

Presence of at least two of these criteria which show inflamatory response to a variety of clinical severe aggressions: temperature above 38°C or below 36°C, heart beat above 90 bpm, respiratory rate above 20rpm or arterial carbon dioxide (PCO2) bellow 32mmHg, leukocytosis over 12,000/mm [3] or leukopenia under 4000/mm [3] or more than 10% of immature forms [22].

#### Quick sequential organ failure assesment (qSOFA)

Two or more of the variables will have to be fulfilled apart from a suspected infection: respiratory rate ≥ 22bpm, Glasgow Coma Scale ≤ 13, Systolic arterial pressure ≤ 100mmHg. No laboratory results are needed. It is a rapid and remeasurable scale [2, 5, 7].

#### National early warning score 2 (NEWS2)

Quantitative scale evaluating heart rate, oxygen saturation, use of supplementary oxygen, temperature (including hypothermia), systolic blood pressure, heart rate and conscious level (A: alert, C: confusion, V: responsive to voice, P: responsive to pain, U: unresponsive), were NEWS2 ≥ 5 can pull the trigger in a physician about the dark prognostic of his patient as shown in different studies [11, 13, 14, 23–29]. Depending on the value of the variable, a score will be given (from 0 to 3), which when added up with the rest of the variables, an overall NEWS2 score is given.

#### Sequential organ failure assesment (SOFA)

Scale for evaluation of organ failure in suspected infection patients estimating respiratory function (PaO2/FiO2), coagulation (platelets), hepatic (bilirubin), cardiovascular (MAP or its manage), neurological (Glasgow scale) and renal (creatinine) systems. The SOFA score will be considered 0 if there is no organic damage evidence previously known in the patient [30]. Each system evaluated will have a score from 0 to 4, that when added up will give the overall SOFA score.

### Amplified risk scales

Based in the present study (see below), we have added three variables which gave statistical signifficane as demonstrated by logistic regression in comparision to other recorded variables, to the qSOFA and NEWS2 scales: PaCO2 ≤ 35mmHg, lactate ≥ 2 mg/dl y glucose blood level ≥ 190 mg/dl. Therefore, we considers cut-oof points ≥ 4 amplified qSOFA (qSOFA-amp) and ≥ 6 amplified NEWS2 (NEWS2-amp).

Due to the sometimes lack of variables recorded, there were cases were the risk scale could not be calculated in all the study population, as shown on the following flow chart, Fig. 1.

### Statistical analysis

Mean and standard deviations for quantitative variables and total number and percentage for qualitative variables have been calculated. Terciles of age were a decided as a post-hoc decision, depending on the patients age distribution. Laboratory variables were treated as linear due to normal distribution as assessed by the Kolmogorov-Smirnov test. ANOVA was used for comparison of quantitative and Chi [2] for qualitative variables. The prognostic value of in-hospital mortality was examined for each variable calculating relative risk and 95% confidence interval. Odds ratio (OR) of mortality was calculated for each scale, the basic and the amplified with more variables, by using logistic regression. The prognostic value for in-hospital mortality of the scales was analyzed by ROC curves. The ROC curves were compared on a cuantitative and cualitative terms. Sensibility, specificity and positive and negative predictive values have been calculated. *p* value <0.05 was considered significant. Statistical analysis was performed by using SPSS pack (IBM SPSS Statistics 24).

## Results

### Characteristics of the study population

We included 201 patients, 54% male, average age of 77±11,2 years old. Sixty-three (31.5%) patients died during hospitalization. The main features of the study population, grouped by mortality in-hospital and three months after discharge, are shown in Table 1. In-hospital mortality was greater in the oldest patients 81 vs 72 years old, (*p*=0,029) and higher dependence (Barthel Index ≤ 35) in basic life activities (*p*=0,029). Although mortality was higher in males as compared to females, no statistical significance was achieved. The sepsis origin was recorded in the cases were infection source was verified during the hospitalization of the patient. The most common source was urinary (51,2%), followed by respiratory (28%), both of them leading the in-hospital mortality source of infection, as shown on Table 1. As a general picture of the CAS causing organisms, 32% were gram-negative bacteria, obtained by blood, urinary or sputum culture, as shown on Table 1. Although, in relation to mortality, these were not satatisticaly significant. As a general picture of CAS causing organisms we founded: Escherichia Coli, Pseudomonna Aeruginosa, Bacterioides Fragilis, Klebsiella Pneumonie, Proteus Mirabilis … Beside gram-negative, we also discovered Staphylococcus spp, Enterococcus spp, Sterptococcus spp.

**Table 1:** General features

	**Total** **(%)**	**Alive** **(%)**	**In-Hospital Mortality (%)**	**Mortality in** 3 Months (%)**	***p*** **value**^**6**^
**TOTAL** (201)*	201 (100,0)	113 (56,5)	63 (31,5)	24 (12,0)	
**GENDER (male)** (201)*	108 (53,7)	55 (27,3)	34 (16,9)	19 (9,5)	**NS**
**AGE (SD)** (201)*	77 (11,9)	72,7 (10,9)	81,3 (12,3)	81,2 (11,3)	**0,029**
**TERCILES OF AGE**					
Group 1 (15 to 74 years old)	80 (39,8)	58 (28,9)	15 (7,5)	7 (3,5)	
Mean ± SD	65,2 ± 8,2	65,3 ± 7,8	64,1 ± 9,9	67,1 ± 8,3	
Group 2 (76 to 84 years old)	60 (29,9)	37 (18,4)	18 (9,0)	5 (2,5)	
Mean ± SD	79,9 ± 3,0	79,9 ± 3,1	79,6 ± 2,8	80,8 ± 3,8	
Groupo 3 (≥ 85 years old)	61 (30,3)	18 (9,0)	30 (14,9)	12 (6,0)	
Mean ± SD	89,8 ± 4,16	88,3 ± 3,54	90,97 ± 4,16	89,5 ± 4,58	
**DIABETES** (201)*	74 (37,0)	41 (20,5)	26 (13,0)	7 (3,5)	**NS**
**DEPENDENCE LEVEL** (195)*					**<0,001**
-TOTAL	69 (35,4)	23 (11,8)	35 (17,9)	11 (5,6)	
-PARTIAL	32 (16,4)	18 (9,2)	11 (5,6)	3 (1,5)	
-INDEPENDENT	94 (48,2)	69 (3,4)	16 (8,2)	9 (4,6)	
**INFECTION SOURCE** (200)*					**NS**
Respiratory	56 (28,0)	27 (13,4)	20 (10,0)	9 (4,5)	
Urinary	102 (51,2)	64 (32,0)	26 (13,0)	12 (6,0)	
Abdominal	19 (9,5)	11 (5,5)	6 (3,0)	2 (1,0)	
Soft tissue	9 (4,5)	5 (2,5)	4 (2,0)	0 (0,0)	
Urinary and respiratory	3 (1,5)	0 (0,0)	2 (1,0)	1 (0,5)	
Mucositis	1 (0,5)	1 (0,5)	0 (0,0)	0 (0,0)	
Arthritic	1 (0,5)	1 (0,5)	0 (0,0)	0 (0,0)	
Unknown	9 (4,5)	4 (2,0)	5 (2,5)	0 (0,0)	
**GRAM-NEGATIVE** (150)*	44 (32,0)	33 (22,0)	11 (7,3)	4 (2,7)	NS
**VITAL SIGNS:**					
**TEMPERATURE** (193)*	37,2 ± 13,0	37,5 ± 1,4	36,9 ± 1,6	36.9 ± 1,2	**0,021**
**SaO2 mmHg** (192)*	91,9 ± 6,1	94,0 ± 5,0	90,0 ± 6,0	89,0 ± 9,0	**0,011**
**FiO2 mmHg** (192)*	0,26 ± 0,1	0,24 ± 0,11	0,29 ± 0,17	0,28 ± 0,14	**0,016**
**SYSTOLIC BP (mmHg)** (199)*	107± 29	109 ± 29	102 ± 30	109,0 ± 28,0	**NS**
**DYASTOLIC BP (mmHg)** (199)*	61 ± 18	62 ± 18	61 ± 19	60 ± 17	**NS**
**HEART RATE (beats/min)** (193)*	103 ± 25	101 ± 22	109 ± 29	98 ± 25	**0,034**
**RESPIRATORY RATE ≥ 22 (resp/min)** (136)*	72 (52,9)	33 (24,3)	29 (21,32)	10 (5,6)	**0,013**
**GLASGOW scale ≤ 13** (180)*	64 (35,6)	20 (11,1)	34 (18,9)	10 (5,6)	**<0,001**
**OTHER:**					
**ICU need** (200)*	28 (14,0)	13 (6,5)	15 (7,5)	15 (7,5)	**0,009**
**Days ICU** (200)*	0,5 ± 1,6	0,0 ± 1,0	1,0 ± 2,0	0,0 ± 0,0	**0,041**
**Days HOSPITAL** (199)*	9 ± 11	12 ± 14	5 ± 4	9 ± 5	**<0,001**

*Number of registered cases, non including lost cases, over total ‘n’ of 201. **Mortality in 3 months represents additional mortality beyond the inpatient mortality ^6^p value comparing alive and hospital mortality. (): Porcentages over n of each cualitative variable. Media ± standard deviation in cuantitative variables

In the supplementary Table 1, the characteristics of the patients did not included in the study due to missing data is presented. In Table 2 is shown that patients who died during hospitalization had lower PaCO2 levels, and higher lactate, glucose and creatinine. Furthermore, patients who were admitted to Intensive Care Unit had the highest mortality. Twenty-four (12%) patients died 3 months after hospital discharge. They were older, 81.2 year, and with higher basic life activity dependence, 58% with dependence.

**Table 2:** Laboratory results versus in-hospital mortality

	**Total** **(%)**	**Alive** **(%)**	**In-Hospital Mortality (%)**	**Mortality in** 3 Months (%)**	***p*** **value**^**6**^
**GASOMETRY**					
**paCO2 mmHg** (158)*	36,2 ± 8,7	37,4 ± 8,0	33,3 ± 8,7	38,8 ± 10,4	**0,004**
**paO2 mmHg** (101)*	64,1 ± 21	62,6 ± 14,2	66 ± 28,5	63,7 ± 19,6	**NS**
**paO2/FiO2** (100)*	261,40 ± 94,00	275,30 ± 71,50	240,19 ± 111,85	263,4 ± 110,6	**NS**
**PH** (179)*	7,40 ± 0,10	7,43 ± 0,06	7,40 ± 0,10	7,41 ± 0,09	**NS**
**CELLS BLOOD COUNT:**					
**WBCs x10**^**3**^**/ml**(200)*	15,84 ± 10,38	16,08 ± 10,89	16,33 ± 10,31	13,44 ± 8,15	**NS**
**Neutrofils x10**^**3**^**/ml** (200)*	13,82 ± 9,97	14,14 ± 10,43	14,33 ± 9,88	11,07 ± 7,89	**NS**
**Platelets x10**^**3**^**/ml (**(198)*	235 ± 128	230 ± 119	248 ± 153	219 ± 101	**NS**
**BLOOD LEVELS**					
**Glucose (mg/dl)** (199)*	170 ± 100	164 ± 92	192 ± 119	144 ± 75	**0,043**
**Creatinine (mg/dl)** (200)*	1,90 ± 1,30	1,67 ± 1,15	2,21 ± 1,31	2,42 ± 13,40	**0,032**
**Bilirrubine (mg/dl)** (131)*	0,80 ± 1,90	0,71 ± 1,21	1,07 ± 3,10	0,68 ± 0,93	**NS**
**Lactate (mEq/L)** (174)*	3,0 ± 2,6	2,4 ± 1,8	4,2 ± 3,3	2,6 ± 2,1	**<0,001**
**Procalcitonin (microg/L)** (104)*	15,36 ± 26,85	14,88 ± 26,02	16,80 ± 28,53	4,80 ± 4,80	**NS**
**CRP (mg/L)** (198)*	116,42 ± 133,3	165,04 ± 142,06	175,39 ± 125’07	142,27 ± 107,13	**NS**

*Number of registered cases, non including lost cases, over total ‘n’ of 201

**Mortality in 3 months represents additional mortality beyond the inpatient mortality

^**6**^*p* value comparing alive and hospital mortality

(): Porcentages over n of each cualitative variable

Media ± standard deviation in cuantitative variables

### In-hospital mortality related factors

Vital signs recorded in triage were related with in-hospital mortality adjusted by age and sex using logistic regression analysis: Glasgow Coma Scale <13 (*p* value < 0,001), respiratory rate >22 bpm (*p*=0,05), temperature (*p*=0,07, NS), oxygen desaturation (*p* =0,074), high flow oxygen therapy (*p*= 0,029) and high recorded heart rate (*p*=0,005). Blood pressure levels, especially hypotension, were not related with a worse prognosis. Other previously studied parameters, such as C-reactive protein (CRP), procalcitonin or leukocytosis were not statistically significant. Although bilirubin levels were superior in in-hospital mortality cases, no significant statistical value was observed. Every risk scale score was recorded at the moment of triaje and blood sample extraction, therefore, during the beginning of the patients stay in the ED.

The achievement of the SOFA OR 2,80; 95% CI 1,12-7,82, NEWS2 OR 7,37; 95% CI 2,51-21,61 and qSOFA OR 3,72; 95% CI 1,81-7,65 were related to a higher in-hospital mortality, as shown in Table 3. On the other hand, amplified scales also demonstrated statistical significant results: qSOFA-amp OR 6; 95% CI 2,47-14,58. NEWS2-amp OR 8,69; 95% CI 2,97-25,41. However, SIRS criteria was the only scale whithout significant differences between groups.

**Table 3:** Risk scales

	**TOTAL** **(%)**	**ALIVE** **(%)**	**IN-HOSPITAL MORTALITY (%)**	**MORTALITY IN** 3 MONTHS (%)**	***p*** **value**^**6**^
**TOTAL** (201)*	201 (100,0)	113 (56,5)	63 (31,5)	24 (12,0)	
**SCALES**					
SIRS ≥ 2 (179)*	149 (83,2)	82 (45,8)	51 (28,5)	16 (8,9)	**NS**
Quantitative SIRS (179)*	2,4 ± 0,9	2,0 ± 1,0	3,0 ± 1,0	2,0 ±1,0	**NS**
qSOFA ≥ 2 (158)*	63 (39,9)	24 (15,2)	28 (17,7)	11 (6,9)	**<0,001**
Quantitative qSOFA (158)*	1,3 ± 0,9	1,0 ± 1,0	2,0 ± 1,0	1,0 ± 1,0	**0,013**
SOFA ≥ 2 (186)*	153 (82,3)	82 (44,1)	51 (27,4)	20 (10,8)	**0,046**
Quantitative SOFA (186)*	3,4 ± 2,4	3,0 ± 2,0	5,0 ± 3,0	4,0 ± 2,0	**<0,001**
NEWS2 ≥ 5 (193)*	150 (77,7)	78 (40,4)	55 (28,5)	17 (8,8)	**<0,001**
Quantitative NEWS2 (193)*	7,5 ± 3,8	6,0 ± 3,0	10,0 ± 3,0	8,0 ± 4,0	**<0,001**
**AMPLIFIED (amp) SCALES**					
qSOFA-amp ≥ 4 (126)*	31 (24,6)	12 (9,5)	17 (11,9)	2 (1,6)	**<0,001**
Quantitative QSOFA-amp (126)*	2,5 ± 1,6	2,0 ± 1,0	3,0 ± 2,0	2,0 ± 1,0	**<0,001**
NEWS2-amp ≥ 6 (193)*	139 (72,0)	62 (32,1)	57 (29,5)	20 (10,4)	**<0,001**
Quantitative NEWS2-amp (193)*	8,6 ± 4,2	7,0 ± 4,0	11,0 ± 4,0	9,0 ± 4,0	**<0,001**

*Number of registered cases, non including lost cases, over total ‘n’ of 201. **Mortality in 3 months represents additional mortality beyond the inpatient mortality ^6^*p* value comparing alive and hospital mortality. (): Porcentages over n of each cualitative variable. Media ± standard deviation in cuantitative variables

### Predictive value of hospital mortality

Sensibility, specificity, predictive positive and negative value for each scale is in Table 4. Predictive value of each scale was tested by using ROC curve. The cut-off points of each new variable included in the amplified scales were calculated selecting the best for each parameters: lactate>2 mg/dl, glucose blood level >190 mg/dl (75 percentile) and PaCO2 <35mmHg. We did not include glucose level of 155 mg/dl as a cut-off point obtained by the Youden Index, since this value is considered low, poorly specific or non representative for septic patients. Variables that demonstrated prognostic value (lactate, glucose blood level and PaCO2) were included for amplified scale

**Table 4:** Sensitivity, specificity and positive and negative prediction value

	**Cut-off values**	**ROC curve** **[IC95%]**	**Sensitivity [IC95%]**	**Specificity [IC95%]**	**PPV** **[IC95%]**	**NPV** **[IC95%]**
QSOFA	≥2	[0,48–0,72]	0,62 [0,48-0,76]	0,70 [0,61-0,78]	0,45 [0,33-0,58]	0,82 [0,75-0,90]
qSOFA amp	≥4	[0,56–0,80]	0,52 [0,35-0,69]	0,85 [0,78-0,92]	0,55 [0,37-0,72]	0,83 [0,76-0.91]
NEWS2	≥5	[0,51–0,72]	0,93 [0,87-0,99]	0,34 [0,26-0,42]	0,40 [0,32-0,48]	0,92 [0,84-0,99]
NEWS2amp	≥6	[0,54–0,74]	0,93 [0,87-0,99]	0,38 [0,30-0,46]	0,41 [0,33-0,49]	0,93 [0,85-0,99]

**Fig. 3:**
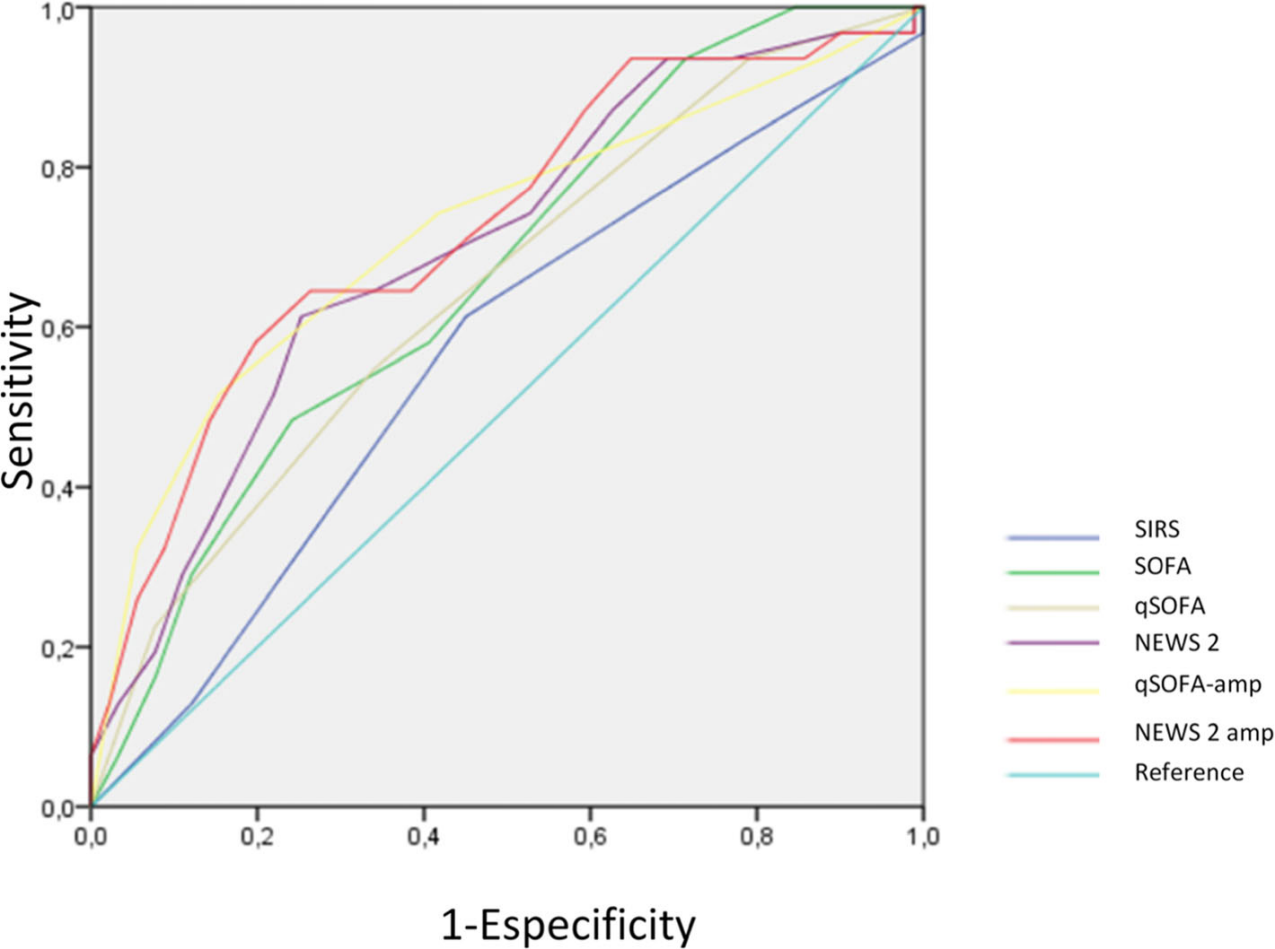
ROC curve for in-hospital mortality according of each scale

Results of the ROC curve for scales are shown in Tables 5 and 6. The cut-off point for a cualitative manner established the limit of signification of each one (Table 4) was calculated to assess the prognostic value of the scales. SIRS ≥ 2, SOFA ≥ 2, qSOFA ≥ 2 and NEWS2 ≥ 5. The cut-off points of the amplified scales were ≥ 4 for amplified qSOFA and ≥ 6 for amplified NEWS2 (Table 4). The greater predictive capacity and area below the curve from the higher to the lower was qSOFA, NEWS2, SOFA and finally SIRS. Amplified scales had an even greater area below their curve (Fig. 3) despite that the number of subjects analyzed for qSOFA amplified are lower that for the initial qSOFA..

**Table 5:** Areas below ROC curves in cuantitative scales

**Scales**	**Area ± SD**	**IC 95%**	**Asintotic Signification**
**SCALES**			
**SIRS**	0,566 ± 0,060	[0,449 - 0,683]	0,274
**SOFA**	0,666 ± 0,054	[0,560 – 0,771]	0,006
**qSOFA**	0,647 ± 0,057	[0,536 – 0,759]	0,015
**NEWS2**	0,699 ± 0,055	[0,592 – 0,806]	0,001
**AMPLIFIED (AMP) SCALES**			
**qSOFA-amp**	0,719 ± 0,058	[0,606 – 0,832]	**<0,001**
**NEWS2-amp**	0,727 ± 0,054	[0,620 – 0,833]	**<0,001**

**Table 6:** Areas below ROC curves in cualitative scales

**SCALES**	**AREA ± SD**	**IC 95%**	**ASINTOTIC SIGNIFICATION**
**SCALES**			
**SIRS ≥ 2**	0,517 ± 0,060	[0,400 – 0,635]	0,778
**SOFA ≥ 2**	0,572 ± 0,057	[0,460 – 0,684]	0,233
**qSOFA ≥ 2**	0,602 ± 0,060	[0,484 – 0,719]	0,093
**NEWS2 ≥ 5**	0,617 ± 0,054	[0,511 – 0,724]	0,053
**AMPLIFIED (AMP) SCALES**			
**qSOFA-amp ≥ 2**	0,586 ± 0,057	[0,474 – 0,698]	0,156
**qSOFA-amp ≥ 3**	0,614 ± 0,056	[0,530 – 0,752]	0,020
**qSOFA-amp ≥ 4**	**0,678 ± 0,060**	[0,560 – 0,795]	**0,003**
**qSOFA-amp ≥ 5**	0,633 ± 0,063	[0,509 – 0,756]	0,029
**NEWS2-amp ≥ 5**	0,565 ± 0,057	[0,453 – 0,678]	0,280
**NEWS2-amp ≥ 6**	**0,640 ± 0,053**	[0,536 – 0,744]	**0,021**
**NEWS2-amp ≥ 7**	0,637 ± 0,054	[0,530 – 0,743]	0,024
**NEWS2-amp ≥ 8**	0,623 ± 0,057	[0,512 – 0,734]	0,043

## Discussion

In this community-acquired sepsis study, some of risk scales and parameters collected in triage at admission have useful prognostic value for mortality although with uneven results. Whereas qSOFA and NEWS2 had a good prognostic value, SIRS criteria was the worst one. Moreover, parameters such as lactate, glucose and PaCO2 increased the prognostic value. The inclusion of these parameters in the qSOFA and NEWS2 scales enhanced their prognostic value. Amplified-qSOFA and amplified-NEWS2 may be usefool tools for early sepsis diagnosis.

Sepsis is still a high incidence illness with difficult management and diagnosis at Emergency Department, were fast decissions are required in a stressful and overcharged atmosphere. In addition, there is a lack of consensus about the accuracy of sepsis diagnosis of the different scales. Even not developed initially to assess prognosis, several studies tried to identify their prognostic value. Previous studies which have examined the prognostic value of the used scales were performed in different settings, such as critically ill [16], surgical [17], cirrhosis [18], oncologic [19]. However, only a few of these studies were carried in CAS sepsis [4]. The present study has been performed at an Emergency Department in a Tertiary Hospital, and the used scales gave the possibility to compare their predictive value. Furthermore, in order to improve their predictive value, we included modifications in the best considered and with higher applicability scales. The high mortality observed may be due to the aged study population. Consider that the University Hospital is located in a urban area were the age-distribution of population is toward large prevalence of elderly people.

The performance of the different scales largely differs and no consensus exists about their use. SIRS scale has lost popularity due to the low specificity and predictive value. The results obtained about the small predictive value of SIRS match with previous studies [5–15]. qSOFA is becoming less used due to the lack of sensitivity although has high specificity [7, 8]. The SOFA scale, despite its high predictive capacity, sensitivity and specificity, it is not the most suitable scale to be used at Emergency Room, due to the necessity of laboratory results and the delay that this requires. This is the reason why SOFA scale is considered as an Intensive Care Unit scale [2, 5, 22]. Finally, according to the last NEWS2 review [12, 15], this scale has a great predictive value for the prognostic of in-hospital mortality risk patients and achieves better results when combined with high lactate levels.

In addition to the scales, we analyzed individual parameters obtained at the Triage showing that some of these had significant predictive value for the in-hospital mortality. Parameters that showed significacy (lactate, glucose blood level and PaCO2) are generally quickly accessible at Emergency Department. Inclusion of these variables can improve the applicability, sensitivity and specificity of the two most practical and better considered Emergency Room scales: qSOFA and NEWS2. The new ROC curves for the amplified scales not only improved the area below the curve, but also outperformed the results of the scale with the best prognostic value, NEWS2.

Our study needs to be contemplated in their straights and limitations. Data were collected from the Electronic Medical Record in which all the information and time-delivered procedures are recorded. Although the sample size is not large and the power calculation has not been assessed before the analysis, it was enough to perform a detailed statistical approach searching for the best parameters and/or scales to assess prognosis in septic patients. Limitations included the lack of testing in an independent cohort and that the amplified scores have better AUC, although we should consider as a limitation the fact that AUC 95% CIs overlap in certain manner with their non-amplified comparators. Likewise, the fact that some data was missed when variables were recorded and therefore could not be included in the scale analysis may have slightly weaken but not threated this study. Obviously a bigger cohort and complex study is required to modify the nowadays used risk scales. Moreover, it was performed at the same Department, including consecutive patients to avoid a selection bias in the results but this results cannot be applied to others clinical settings.

## Conclusion

In conclusion, amplified-qSOFA and amplified-NEWS2 with these three parameters, lactate, glucose and PCO2, may be useful tools for early sepsis diagnosis at Emergency Department and could increase the mortality prognostic value offered by the available scales. Also, we should consider that these scales could be combined with electronically triggered monitorisation in order to alert clinicians and therefore achieve a quicker response to patient deterioration, as shown in various studies nowadays [31–34]. This could evolve to an improvement in anticipation and subsequently to a better prognosis. Validation studies are needed to verify the prognostic value observed and to evaluate the usefulness of their implementation to reduce mortality in septic patients.

## Supplementary Information

**Additional file 1.** Supplementary Table 1. Total patients and missing variables in the study population

## Data Availability

The datasets used and/or analysed during the current study available from the corresponding author on reasonable request.

## References

[1] Azkárate, I., Sebastián, R., Cabarcos, E., Choperena, G., Pascal, M., Salas, E. (2011). Registro observacional y prospectivo de sepsis grave/ shock séptico en un hospital terciario de la provincia de Guipúzcoa. Med Intensiva, 36:250–6. 10.1016/j.medin.2011.10.006 22154280

[2] Napolitano LM (2018). Sepsis 2018: Definitions and guideline changes. Surg Infect.

[3] Chaplin S (2016). NICE guidance on the diagnosis and early management of sepsis. Prescriber.

[4] Tusgul S, Carron P, Yersin B, Calandra T, Dami F (2017). Low sensitivity of qSOFA, SIRS criteria and sepsis definition to identify infected patients at risk of complication in the prehospital setting and at the emergency department triage. Scand J Trauma Resuscitation Emerg Med.

[5] Rhee C, Klompas M (2017). New sepsis and septic shock definitions: Clinical implications and controversies. Infect Dis Clin N Am.

[6] Giamarellos-Bourboulis EJ, Tsaganos T, Tsangaris I, Lada M, Routsi C, Sinapidis D, et al. Validation of the new sepsis-3 definitions: Proposal for improvement in early risk identification. Clin Microbiol Infect. 2017;23:104–9. 10.1016/j.cmi.2016.11.003 27856268

[7] Sartelli M, Kluger Y, Ansaloni L, Hardcastle TC, Rello J, Watkins RR, et al. Raising concerns about sepsis-3 definitions. World J Emerg Surg. 2018;13:6-27. 10.1186/s13017-018-0165-6 PMC5784683 29416555

[8] Maitra S, Som A, Bhattacharjee S (2018). Accuracy of quick sequential organ failure assessment (qSOFA) score and systemic inflammatory response syndrome (SIRS) criteria for predicting mortality in hospitalized patients with suspected infection: A meta-analysis of observational studies. Clin Microbiol Infect.

[9] Vincent JL, Sakr Y, Sprung CL, Ranieri VM, Reinhart K, Gerlach H, Moreno R, Carlet J, Le Gall JR, Payen D (2006). Sepsis in European intensive care units: Results of the SOAP study. Crit Care Med.

[10] Martin GS (2012). Sepsis, severe sepsis and septic shock: Changes in incidence, pathogens and outcomes. Expert Rev Anti-Infect Ther.

[11] Keep J, Messmer A, Sladden R, Burrell N, Pinate R, Tunnicliff M, Glucksman E (2016). National early warning score at emergency department triage may allow earlier identification of patients with severe sepsis and septic shock: A retrospective observational study. Emerg Med J.

[12] Hargreaves D, de Carvalho JL, Smith L, Picton G, Venn R, Hodgson L (2020). Persistently elevated early warning scores and lactate identifies patients at high risk of mortality in suspected sepsis. Eur J Emerg Med.

[13] Silcock D, Corfield A, Staines H, Rooney K (2019). Superior performance of national early warning score compared with quick sepsis-related organ failure assessment score in predicting adverse outcomes: A retrospective observational study of patients in the prehospital setting. Eur J Emerg Med.

[14] Mellhammar L, Linder A, Tverring J, Christensson B, Boyd JH, Sendi P, Akesson P, Kahn F (2019). NEWS2 is superior to qSOFA in detecting sepsis with organ dysfunction in the emergency department. J Clin Med.

[15] Shamout F, Zhu T, Clifton L, Briggs J, Prytherch D, Meredith P, et al. Early warning score adjusted for age to predict the composite outcome of mortality, cardiac arrest or unplanned intensive care unit admission using observational vital-sign data: a multicentre development and validation. BMJ Open. 2019;9(11):e033301. 10.1136/bmjopen-2019-033301 PMC6887005 31748313

[16] Zhang Y, Luo H, Wang H, Zheng Z, Ooi OC (2020). Validation of prognostic accuracy of the SOFA score, SIRS criteria, and qSOFA score for in-hospital mortality among cardiac-, thoracic-, and vascular-surgery patients admitted to a cardiothoracic intensive care unit. J Card Surg.

[17] Green SL, Smith MTD, Cairns C, Clarke DL, Bruce J, Bekker W, Kong V, Laing GL (2020). The combined SIRS + qSOFA (qSIRS) score is more accurate than qSOFA alone in predicting mortality in patients with surgical sepsis in an LMIC emergency department. World J Surg.

[18] Augustinho FC, Zocche TL, Borgonovo A, Maggi DC, Rateke ECM, Matiollo C, Dantas-Correa EB, Narciso-Schiavon JL, Schiavon LL (2019). Applicability of Sepsis-3 criteria and quick Sequential Organ Failure Assessment in patients with cirrhosis hospitalised for bacterial infections. Liver Int.

[19] Costa RT, Nassar AP, Caruso P (2018). Accuracy of SOFA, qSOFA, and SIRS scores for mortality in cancer patients admitted to an intensive care unit with suspected infection. J Crit Care.

[20] Boillat-Blanco N, Mbarack Z, Samaka J, Mlaganile T, Mamin A, Genton B, Kaiser L, Calandra T, D'Acremont V (2018). Prognostic value of quickSOFA as a predictor of 28-day mortality among febrile adult patients presenting to emergency departments in dar es salaam, tanzania. PloS One.

[21] Park HK, Kim WY, Kim MC, Jung W, Ko BS (2017). Quick sequential organ failure assessment compared to systemic inflammatory response syndrome for predicting sepsis in emergency department. J Crit Care.

[22] Cabrita A, Pinheiro J, Falcão LM (2018). Rethinking the concept of sepsis and septic shock. Eur J Inter Med.

[23] Brink A, Alsma J, Verdonschot R, Rood P, Zietse B, Lingsma H, Klein Nagelvoort-Schuit S (2019). Predicting mortality in patients with suspected sepsis at the emergency department; A retrospective cohort study comparing qSOFA, SIRS and national early warning score. PLoS One.

[24] Churpek MM, Snyder A, Han X, Sokol S, Pettit N, Howell MD, Edelson DP (2017). Quick sepsis-related organ failure assessment, systemic inflammatory response syndrome, and early warning scores for detecting clinical deterioration in infected patients outside the intensive care unit. Am J Respir Crit Care Med.

[25] Hamilton F, Arnold D, Baird A, Albur M, Whiting P (2018). Early warning scores do not accurately predict mortality in sepsis: A meta-analysis and systematic review of the literature. J Infect.

[26] Kangas C, Iverson L, Pierce D. Sepsis Screening: Combining Early Warning Scores and SIRS Criteria. Clin Nurs Res. 2021;30:42-49. 10.1177/1054773818823334 30654646

[27] Goulden R, Hoyle M, Monis J, Railton D, Riley V, Martin P, Martina R, Nsutebu E (2018). qSOFA, SIRS and NEWS for predicting inhospital mortality and ICU admission in emergency admissions treated as sepsis. Emerg Med J.

[28] Steele L, Hill S (2019). Using sepsis scores in emergency department and ward patients. Br J Hosp Med.

[29] Usman OA, Usman AA, Ward MA (2019). Comparison of SIRS, qSOFA, and NEWS for the early identification of sepsis in the emergency department. Am J Emerg Med.

[30] Singer M, Deutschman CS, Seymour CW, Shankar-Hari M, Annane D, Bauer M, Bellomo R, Bernard GR, Chiche JD, Coopersmith CM, Hotchkiss RS, Levy MM, Marshall JC, Martin GS, Opal SM, Rubenfeld GD, van der Poll T, Vincent JL, Angus DC (2016). The third international consensus definitions for sepsis and septic shock (sepsis-3). JAMA.

[31] Downey CL, Croft J, Ainsworth G, Buckley H, Shinkins B, Randell R, et al. Trial of remote continuous versus intermittent NEWS monitoring after major surgery (TRaCINg): a feasibility randomised controlled trial. Pilot Feasibility Stud. 2020 23:183-92. 10.1186/s40814-020-00709-8 PMC7684886 33292669

[32] Posthuma LM, Downey C, Visscher MJ, Ghazali DA, Joshi M, Ashrafian H, et al. Remote wireless vital signs monitoring on the ward for early detection of deteriorating patients: A case series. Int J Nurs Stud. 2020;104:103515. 10.1016/j.ijnurstu.2019.103515 32105974

[33] Sa MB, Gonzalez FJC, Roca RF, Cortes PV, Crespo RZ. [Código Sepsis. Documento de Consenso]. IMC – SA. 2014, pp 3-23 [spanish] www.codigosepsis.com.

[34] Martino IF, Figgiaconi V, Seminari E, Muzzi A, Corbella M, Perlini S (2018). The role of qSOFA compared to other prognostic scores in septic patients upon admission to the emergency department. Eur J Intern Med.

