# Supplementary material for: Prognostic value of diagnostic scales in community-acquired sepsis mortality at an emergency service. Prognosis in community-adquired sepsis
Source: BMC Emerg Med. 2021 Dec 18;21:161. doi: 10.1186/s12873-021-00532-1 (PMC8684687; doi:10.1186/s12873-021-00532-1)
Supplement: Supplementary file 1 — Additional file 1. Supplementary Table 1. Total patients and missing variables in the study population [file 12873_2021_532_MOESM1_ESM.docx]

**Supplementary Table 1. Total patients and missing variables in the study population.**

|  | **Total (%)** | **QSOFA**  **missing data (%)**** | **NEWS2**  **missing data (%)**** |
| --- | --- | --- | --- |
| **TOTAL** (201)* | 201 (100,0) | 43 (21,4) | 8 (0,04) |
| **GENDER (male)** (201)* | 108 (53,7) | 26 (60,4) | 4 (50,0) |
| **AGE (SD)** (201)* | 77 (11,9) | 77 (2,0) | 70 (2,0) |
| **DIABETES** (201)* | 74 (37,0) | 12**(*)** (28,0) | 5 (62,5) |
| **DEPENDENCE LEVEL** (195)* |  |  |  |
| -TOTAL | 69 (35,4) | 15 (34,9) | 0 (0,0) |
| -PARTIAL | 32 (16,4) | 3 (7,0) | 2 (25,0) |
| -INDEPENDENT | 94 (48,2) | 21 (48,8) | 5 (62,5) |
| **INFECTION SOURCE** (200)* |  |  |  |
| Respiratory | 56 (28,0) | 9 (20,9) | 1 (12,5) |
| Urinary | 102 (51,2) | 17 (39,5) | 2 (25,0) |
| Abdominal | 19 (9,5) | 5 (11,6) | 1 (12,5) |
| Soft tissue | 9 (4,5) | 3 (7,0) | 0 (0,0) |
| Urinary and respiratory | 3 (1,5) | 1 (2,3) | 0 (0,0) |
| Mucositis | 1 (0,5) | 1 (2,3) | 1 (12,5) |
| Arthritic | 1 (0,5) | 0 (0,0) | 0 (0,0) |
| Unknown | 9 (4,5) | 5 (11,6) | 1 (12,5) |
| **GRAM-NEGATIVE** (150)* | 44 (32,0) | 16 (37,2) | 2 (25,0) |
|  |  |  |  |
| **VITAL SIGNS:** |  |  |  |
| **TEMPERATURE** (193)* | 37,2 ± 13,0 | 37,5 ± 1,5 {3 (7,0)}*** | 38,2 ± 2,4 {1 (12,5)}*** |
| **SaO2 mmHg** (192)* | 91,9 ± 6,1 | 93,0 ± 6,0 {2 (4,7)}*** | 94 ± 3,0 {2 (25,0)}*** |
| **FiO2 mmHg** (192)* | 0,26 ± 0,1 | 0,27 ± 0,15 {1 (2,3)}*** | 0,25 ± 0,08 {3 (37,5)}*** |
| **SYSTOLIC BP (mmHg)** (199)* | 107± 29 | 103 ± 30 {1 (2,3)}*** | 105 ± 26 {1 (12,5)}*** |
| **DYASTOLIC BP (mmHg)** (199)* | 61 ± 18 | 59 ± 15 {1 (2,3)}*** | 54 ± 10 {1 (12,5)}*** |
| **HEART RATE (beats/min)** (193)* | 103 ± 25 | 104 ± 26 {3 (7,0)}*** | 102 ± 31 {1 (12,5)}*** |
| **RESPIRATORY RATE ≥22 (resp/min)** (136)* | 72 (52,9) | 33 (76,7) | 6 (75,0) |
| **GLASGOW scale ≤ 13** (180)* | 64 (35,6) | 16 (37,2) | 2 (25,0) |
|  |  |  |  |
| **OTHER:** |  |  |  |
| **ICU need** (200)* | 28 (14,0) | 7 (16,7) | 3 (37,5) |
| **Days ICU**  (200)* | 0,5 ± 1,6 | 1,0 ± 2,0 | 2,0 ± 3,0 |
| **Days HOSPITAL** (199)* | 9,0 ± 11,0 | 8,0 ± 12,0 | 19,0 ± 30,0 |

***% obtained from total of qSOFA or NEWS2 missing respectively*

*{}***Number of cases (and its following percentage from the total number of missed cases) with the missed variable which therefore could not be included in the scale score analysis*
